# Usefulness of a visual aid in achieving optimal positioning for spinal anesthesia: a randomized trial

**DOI:** 10.1186/s12871-017-0467-3

**Published:** 2018-01-20

**Authors:** Usha Gurunathan, Shakeel Meeran Kunju, Karen Elizabeth Hay, Sharyn van Alphen

**Affiliations:** 1 Department of Anaesthesia and Perfusion services, The Prince Charles Hospital and University of Queensland, Brisbane, Australia; 2 QIMR Berghofer Medical Research Institute, Brisbane, Australia

**Keywords:** Anesthesia, Patient positioning, Anesthesia, Spinal

## Abstract

**Background:**

Optimal patient positioning is perceived as an essential factor to increase the success of performing neuraxial blockade. The primary objective of this study was to evaluate the benefit of using a visual image in addition to verbal instructions in order to optimize positioning for spinal block.

**Methods:**

This was a prospective randomized controlled trial on 85 adult patients undergoing lower limb joint replacements at a tertiary academic hospital. Group 1(*n* = 43) randomized to receive standardized verbal instructions; Group 2 (*n* = 42) received standardized verbal instructions in conjunction with visual aids to achieve positioning for spinal anesthesia. The primary outcome measure was the time taken to successful dural puncture. Secondary endpoints were the number of skin punctures, number of intervertebral levels attempted, success at the first intervertebral space attempted and satisfaction of patients and anesthesiologists.

**Results:**

The unadjusted geometric mean time taken for the procedure using verbal instruction alone was 301 s (95% CI: 236–385) compared to 183 s (95% CI: 143–235) when both verbal and visual instructions were used. Out of the participants in group 2, 90% required ≤2 skin punctures and 10% required ≥3 skin punctures compared to 65% and 35% of the participants in group 1 respectively (*p* = 0.001). Group 1 required a second anesthesiologist to successfully complete the procedure in 6 patients out of 43 (14%) patients whereas the first anesthesiologist was noted to be successful in all the 42 cases in group 2 (*p* = 0.03). There were no significant differences in the satisfaction scores of anesthesiologists or patients between the groups. First-pass success was strongly associated with patient satisfaction (Odds ratio: 5.2; 95% CI: 1.0–9.5, *p* = 0.049).

**Conclusions:**

Use of a visual aid in addition to verbal instructions to optimize positioning for a spinal block, significantly reduces the time taken for the procedure by an average of 2 min, reduces the number of skin punctures and increases the success rate of the first anesthesiologist. First pass success was strongly associated with patient satisfaction.

**Trial registration:**

This study was retrospectively registered 30 August 2016, with the Australian New Zealand Clinical trials registry (ACTRN12616001197426).

**Electronic supplementary material:**

The online version of this article (10.1186/s12871-017-0467-3) contains supplementary material, which is available to authorized users.

## Background

Anesthesiologists traditionally perform neuraxial block utilising landmark- based techniques and the most significant predictor of the difficulty in performing them has been reported to be the quality of these landmarks [1]. Besides landmark quality, patient positioning can also independently predict the success of performing neuraxial anesthesia [2].

Patients are often verbally instructed by the anesthesiologist to adopt an optimal position for neuraxial blocks. Difficulty can be encountered in achieving the best position if patients misunderstand these instructions. This may lead to a delay in performing the procedure and increase the technical difficulty with a possible increased risk of complications [2]. Further, it may result in the anesthesiologist abandoning the procedure despite the possible advantage of neuraxial anesthesia for the patient. Multiple punctures can lower patient satisfaction and may lead to complications such as hematoma, paresthesia and nerve injury as well as post dural puncture headache [1, 3, 4].

Although there have been many studies discussing the predictors for a difficult neuraxial blockade [1, 2, 5], few studies have looked at techniques to minimise these difficulties. Ultrasound has been investigated; however, this technique requires specific training and expertise. One previous study demonstrated that use of visual aids resulted in better positioning in obstetric patients undergoing epidural anesthesia [6]. However, the potential benefits of employing a simple visual aid amongst a more general surgical population remain unexplored.

The aim of our randomized controlled study was to assess the effects of showing patients visual images of the optimal position, in addition to providing verbal instructions, for the placement of spinal anesthesia when compared to providing standardized verbal instructions alone, in adult patients undergoing hip and knee arthroplasty. We hypothesized that the addition of the visual aid would result in improved positioning resulting in reduced time taken for the procedure, improved ease of placement measured by the number of skin punctures required and higher patient satisfaction.

## Methods

The study was approved by the Institutional Human Research and Ethics Committee on the 20th December 2013 (HREC/ 13/QPCH/335). It was retrospectively registered with the Australian New Zealand Clinical trials registry (ACTRN12616001197426). Adult patients undergoing elective hip and knee arthroplasty under spinal anesthesia were included in this prospective randomized controlled study conducted at the Prince Charles Hospital, Brisbane, Australia. Exclusion criteria were refusal to consent, inability to read or comprehend English, any contraindication to neuraxial anesthesia, prior experience with neuraxial blocks, body mass index (BMI) greater than 45 kg/m^2^ and anticipated positioning difficulties due to severe arthritis or major spinal deformities.

Following written informed consent, the eligible participants were randomly allocated into two equal groups, based on computer generated random numbers. Random allocation was achieved by means of double opaque, sealed envelopes marked with trial identification numbers. Blinding was not possible due to the nature of the intervention. Participants in group 1 received standardized verbal instructions only, whereas those in group 2 were additionally shown visual images of the preferred position. The standardized verbal instructions used for all the participants are given in detail (see Additional file 1). These included commonly used phrases, agreed upon after discussion with anesthetic colleagues. The visual images used in this study for the participants in group 2 included photos of one correct position and three incorrect positions. The images intended to convey the same information as the verbal instructions. These images are provided in detail (see Additional file 2).

Following intravenous cannulation and application of standard monitoring, the participants were requested to sit in the upright position on the operating table with their feet resting on a stool, and shoulders gently supported by an assistant. No further assistance in positioning or any verbal prompting was provided by the theatre assistant. No sedative or anxiolytic premedication was administered to the participants, due to possible interference with their comprehension. Anesthesiologists were allowed to choose the type, level, and approach of the block, as well as the needle and local anesthetic used.

The anesthesiologist prepared the spinal tray and drugs under aseptic conditions, and prepared and draped the patient’s back. A second person started the timer and read out the standardized verbal instructions with or without visual aids, according to the allocation group of the patient. The patient then adopted the required position, and the anesthesiologist immediately commenced the procedure. A trained independent observer recorded the relevant data.

The primary outcome measured was the length of time taken until successful needle placement. This was defined as the time in seconds from the start of the instructions until the free flow of cerebrospinal fluid (CSF). Secondary endpoints included number of skin punctures, number of intervertebral spaces that were attempted, success at the first attempt (first pass success), number of anesthesiologists required to successfully complete the procedure and satisfaction scores for both the anesthesiologist and the patient. The number of skin punctures was the total count of all the skin punctures excluding local anesthetic infiltration [7]. The number of intervertebral spaces was the number of spinal levels attempted. Satisfaction scores were assessed on a numerical scale of 1–5 (1: very unsatisfied and 5: very satisfied).

A sample size of approximately 43 patients per group was calculated, based on obtaining 90% power to detect a difference of 2.5 min between average times for the two interventional groups, at a 5% significance level and based on a standard deviation of approximately 3.5 min [8].

The distribution of the primary outcome of interest, time to successful needle placement, was positively skewed so a natural logarithmic transformation was applied to derive a normally distributed outcome variable (log-time) for use in statistical analyses. The exposure of interest was the instruction group (verbal only versus verbal plus visual). Potential confounders of interest included age, sex, BMI, educational level, experience of the first anesthesiologist and the perceived quality of bony landmark.

Differences between the distributions of baseline characteristics across the instruction groups were compared using Pearson’s chi-squared test or Fisher’s exact test for categorical variables or Student’s t-tests for continuous variables. The distribution of time taken was compared across intervention groups using Wilcoxon rank-sum test and linear regression of the log-transformed variable. Linear regression modelling was used to assess associations between the intervention group, potential confounders and log-time. Variables were included in the adjusted multivariable model if it was considered biologically plausible that the variable was a potential confounder of the relationship. Variables remained in the model if inclusion influenced the effect estimate of the intervention.

The secondary outcomes of interest were described using categorical variables. The association between these variables and the type of instruction given were assessed using Pearson’s chi-squared test or Fisher’s exact test where more than 20% of cells had fewer than five observations. Patient satisfaction was collapsed into two categories (dissatisfied/neutral and satisfied) and logistic regression models were fitted to determine the effect estimates for variables associated with patient satisfaction.

All analyses were performed using the Stata statistical software package (version 13) (Statacorp).

## Results

**Fig. 1:**
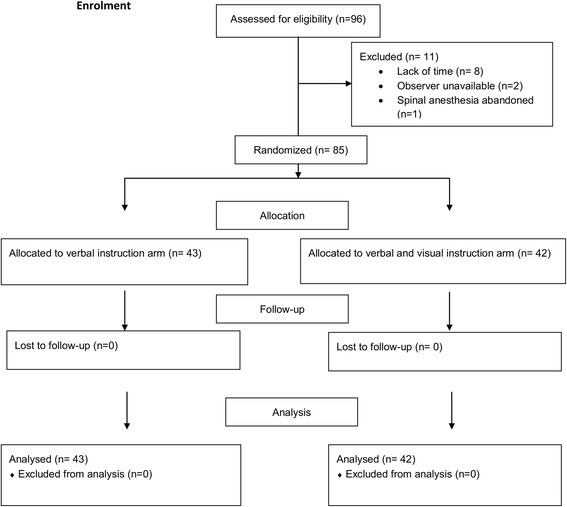
CONSORT flow diagram showing enrolment, allocation, follow up and analysis of the study participants

The study population comprised of 85 patients enrolled from August 1st, 2014 to June 29th, 2016. All the patients that were enrolled for the study were analysed (Fig. 1). Out of the 85 participants, 43 received verbal instructions (Group 1) and 42 received both verbal and visual instructions (Group 2). Patients’ ages ranged from 29 to 89 years (mean: 66; SD 11). The average BMI of the participants was 31 kg/m^2^ (SD: 5). Summary statistics for variables of interest by interventional group are presented in Table 1. The distribution of gender differed significantly across instruction groups but the mean age and BMI did not.

**Table 1:** Baseline characteristics of the patients by instruction group

Variable & category	Verbal (*n* = 43)	Verbal and visual (*n* = 42)	*p*-value
Age in years (mean (SD))	68 (9)	65 (12)	0.102^a^
BMI (mean (SD)) (kg/m^2^)	32 (5)	31 (5)	0.413^a^
Gender (n (%))			0.037^b^
Male	24 (56)	14 (33)	
Female	19 (44)	28 (67)	
Education (n (%))			0.745^b^
Primary	16 (37)	13 (31)	
Secondary	21 (49)	24 (57)	
Tertiary	6 (14)	5 (12)	
Prior spinal education^d^ (n (%))			1.00
Yes	3 (7)	3 (7)	
No	39 (91)	38 (90)	
Experience of anesthesiologist (n (%))			0.351^c^
< 6 months	4 (9)	5 (12)	
6mths- <2 years	3 (7)	1 (2)	
2+ years	32 (74)	27 (64)	
Consultant	4 (9)	9 (21)	
Bony landmarks (n (%))			0.738^b^
Clear	36 (84)	34 (81)	
Unclear	7 (16)	8 (19)	

*SD* standard deviation, *BMI* body mass index

*p*-values derived from: ^a^Student’s t-test; ^b^Pearson’s chi-square test; ^c^Fisher’s exact test

^d^Totals differ due to missing values

Summary statistics for outcome variables by the instruction group are presented in Table 2. The time taken for successful needle placement ranged from 30 to 2053 s. Overall, the median time taken was 227 s (3.8 min); 294 s in the verbal group in comparison to 175 s in the verbal plus visual group (Table 2). The results of univariable and multivariable linear regression of predictors on log-time are presented in Table 3. The instruction group, patient’s BMI and perceived quality of a bony landmark were significantly associated with the time taken in univariable models. In the adjusted model, instruction group remained significantly associated with time after adjusting for BMI, perceived quality of a bony landmark, age and gender. The unadjusted geometric mean time taken for the procedure using verbal instruction alone was 301 s (95% CI: 236–385) compared to 183 s (95%CI: 143–235) when both verbal and visual instructions were used. Thus, procedures were on average about 2 min and 39% shorter using the visual instructions in conjunction with the verbal instructions. Post –hoc calculations based on the log-transformed variables showed that a sample size of 44 per group would achieve 90% power at the 5% significance level to detect a difference between log-transformed mean values of 300 and 150 s, assuming a standard deviation of one for the log-transformed variable. This was consistent with the initially estimated sample size.

**Table 2:** Distribution of outcomes of interest by the intervention group

Variable & category	Verbal (*n* = 43)	Verbal and visual (*n* = 42)	*p*-value
Time taken (median (IQR) (seconds)	294 (155–625)	175 (116–260)	0.008^b^
Number of intervertebral spaces^a^ (n (%))			0.148^c^
1	28 (65)	32 (76)	
2	8 (19)	8 (19)	
≥ 3	6 (14)	1 (2)	
Number of skin punctures^a^ (n (%))			0.001^d^
1	24 (56)	22 (54)	
2	4 (9)	15 (37)	
≥ 3	15 (35)	9 (10)	
Number of anesthesiologists (n (%))			0.026^d^
1	37 (86)	42 (100)	
2	6 (14)	0 (0)	
Anesthesiologist’s satisfaction (n (%))			0.833^c^
Dissatisfied	5 (12)	3 (7)	
Neutral	6 (14)	5 (12)	
Satisfied	16 (37)	19 (45)	
Very satisfied	16 (37)	15 (36)	
Patient satisfaction^a^ (n (%))			0.183^d^
Dissatisfied	1 (2)	1 (2)	
Neutral	6 (14)	1 (2)	
Satisfied	13 (30)	13 (31)	
Very satisfied	20 (47)	26 (62)	
First pass success (n (%))			0.926^c^
Yes	25 (58)	24 (57)	
No	18 (42)	18 (43)	
Successful (n (%))			1.00^d^
Yes	41 (95)	41 (98)	
No	2 (5)	1 (2)	

*IQR* interquartile range

^a^Totals differ due to missing values

*p*-values derived from: ^b^Wilcoxon rank-sum test; ^c^Pearson’s chi-square test; ^d^Fisher’s exact test

**Table 3:** Results of linear regression of variables of interest on time taken to complete procedure (log-transformed)

Variable & category	Unadjusted		Adjusted^a^	
	Coefficient (95% CI)	*p*-value	Coefficient (95% CI)	*p*-value
Instruction				
Verbal	ref		ref	
Verbal and visual	−0.5 (−0.8 to −0.2)	0.006	−0.4 (−0.8 to −0.1)	0.022
BMI per unit (kg/m^2^)	0.05 (0.02 to 0.09)	0.003	0.05 (0.01 to 0.08)	0.007
Bony landmark				
Clear	ref		ref	
Unclear	0.5 (0.1 to 1.0)	0.015	0.42 (−0.0 to 0.9)	0.067

^a^Estimates also adjusted for age and gender

The number of skin punctures and number of anesthesiologists varied significantly by instruction group (Table 2). Out of the participants in group 2, 90% required ≤2 skin punctures and 10% required ≥3 skin punctures compared to 65% and 35% of the participants in group 1 respectively (*p* = 0.001). Group 1 required a second anesthesiologist to successfully complete the procedure in 6 patients out of 43 (14%) patients whereas in all the 42 cases of group 2 (100%), the first anesthesiologist was noted to be successful (*p* = 0.03) (Table 2).

There were no significant differences between groups in the detailed satisfaction scores of anesthesiologists or patients (Table 2); but the majority of patients (89%) reported being satisfied or very satisfied. The results of univariable logistic regression of predictors on patient satisfaction (neutral or dissatisfied compared to satisfied) are presented in Table 4.

**Table 4:** Results of logistic regression of variables associated with patient satisfaction

Variable & category	OR (95% CI)	*p*-value
Instruction group		
Verbal	Ref	
Verbal and visual	4.1 (0.8–21.3)	0.089
First pass success		
No	Ref	
Yes	5.2 (1.0–9.5)	0.049

First-pass success was strongly associated with patient satisfaction (Odds ratio (OR): 5.2; 95% CI: 1.0–9.5). There was also some evidence that instruction group was associated with patient satisfaction (OR: 4.1; 95% CI: 0.8–21.3). However, these estimates were imprecise due to low power for this outcome.

## Discussion

Our study demonstrated that the use of a simple visual aid in conjunction with verbal instructions resulted in improved positioning of patients undergoing spinal anesthesia as measured by significantly reduced time taken for the procedure, improved ease of spinal needle placement with fewer skin punctures and reduced need for assistance from a second more experienced anesthesiologist.

The results in our study are consistent with those of a similar study on obstetric patients undergoing epidural anesthesia for caesarean section in which use of visual image resulted in a reduction in both time taken and number of needle punctures, though not statistically significant at the 5% level. However, our study includes general surgical patients with different demographic and clinical characteristics.

Our observation of the univariable association between the quality of bony landmarks and time taken is consistent with previous studies which have shown that the quality of the anatomical landmarks can significantly predict the degree of difficulty in performing spinal anesthesia [1, 5]. However, when our results were adjusted for BMI, the effect of the quality of bony landmarks was much attenuated, indicating the influence of BMI on the quality of landmarks. Thus, while randomization is expected to balance the effects of potential confounders, in also fitting a multivariable model we have assessed residual confounding, and investigating the effects of other important factors such as BMI and the presence of bony landmarks. Since we excluded patients with major spinal deformities and those with BMI greater than 45 kg/m^2^, although we saw a significant reduction in the number of skin punctures with the visually aided group, we were not able to find any difference in the number of intervertebral levels attempted.

It has been previously shown that three or more attempts at spinal anesthesia, leads to significant patient dissatisfaction [4]. This dissatisfaction with the procedure may increase the risk of patients refusing to have neuraxial blocks in the future [4]. We hypothesized that a shorter time to a successful blockade may improve the satisfaction of the patients and the proceduralist. In our study, we noted that first pass success had a strongly significant association with patient satisfaction. However, our estimate of the magnitude of the effect was imprecise due to the study being underpowered to detect any difference in satisfaction scores.

Our study has some strengths and limitations. To our knowledge, this is the first study to consider the use of a simple and practical aid such as visual images to assist positioning for spinal blocks in a general surgical population. Unlike the previous study [6], we have investigated the effects of possible confounders such as experience of anesthesiologists, educational status of patients and previous experience with neuraxial anesthesia. We excluded patients with any prior neuraxial experience. We also did not attempt to blind the anesthesiologists to the nature of intervention as per earlier study [6], to ensure that we measure the usefulness of our intervention in actual clinical practice and increase external validity of our findings. We decided to investigate its use only in spinal anesthesia (rather than epidural anesthesia), as the free flow of CSF is a definite endpoint that enabled us to measure the time to success with precision [7]. However, we believe that the results should be applicable to any neuraxial anesthesia. Lack of anxiolytic premedication for the study participants could have potentially limited the study’s generalizability. We are unable to draw conclusions on whether showing an image improved the quality of landmarks, as it was not one of our outcomes. Additionally, as the anesthesiologists were not blinded, this could have introduced some bias in ascertaining the clarity of landmarks. We did not analyze the different types or gauges of needles used or the location of punctures (midline/paramedian) as it has been previously shown not to predict the number of attempts [1]. Moreover, the majority of the anesthesiologists at our center choose 25 G Sprotte needle as a default first option. In our study, as the majority (85%) of our anesthesiologists had more than 2 years of anesthetic training we are not able to comment on the impact of the level of experience. However, we speculate that if more junior trainees were included, a bigger difference in the time taken, between the two groups may have resulted.

We are unable to comment on the utility of this approach in improving spinal anesthesia in patients with BMI greater than 45 kg/m^2^ or in patients with difficult spinal anatomy as patients with these characteristics were excluded from our study and may require ultrasound assistance. However, with more than 50% of our participants having a BMI ≥ 30 kg/m^2^, we conclude that a visual aid may also be beneficial to patients with a higher BMI. Although the use of neuraxial ultrasound is on the increase and has proven to be beneficial in patients with anticipated difficulties such as spinal deformities and obese patients, use of the blind landmark based technique is still very popular. The advantage of neuraxial ultrasound in patients without difficult landmark ascertainment is questionable [9]. Hence it becomes important to advocate the use of additional easy and practical measures to improve the success rate of this procedure before neuraxial ultrasound becomes standard of care.

## Conclusion

In summary, we conclude that the use of visual aids to assist positioning for neuraxial blockade reduced the time taken for the procedure by an average of 2 min and also reduced the number of skin punctures required. The use of a visual image also resulted in a 100% success rate for the first anesthesiologist without the need for a second anesthesiologist to intervene. First pass success was strongly associated with patient satisfaction.

## Additional files

Additional file 1: Standardized verbal instructions. Description of the data: Standardized verbal instructions used for all the participants included commonly used phrases, agreed upon after discussion with anesthetic colleagues. (DOCX 35 kb)

Additional file 2: Visual images used for the spinal positioning. Description of the data: The visual images used in the study for the participants in group 2 included photos of one correct position and three incorrect positions. The images intended to convey the same information as the verbal instructions. (TIFF 15913 kb)

## Abbreviations

BMI: Body mass index
CI: Confidence interval
CSF: Cerebrospinal fluid
OR: Odds ratio
