# Supplementary material for: Usefulness of a visual aid in achieving optimal positioning for spinal anesthesia: a randomized trial
Source: BMC Anesthesiol. 2018 Jan 20;18:11. doi: 10.1186/s12871-017-0467-3 (PMC5775525; doi:10.1186/s12871-017-0467-3)
Supplement: Supplementary file 1 — Standardized verbal instructions. Description of the data: Standardized verbal instructions used for all the participants included commonly used phrases, agreed upon after discussion with anesthetic colleagues. (DOCX 35 kb) [file 12871_2017_467_MOESM1_ESM.docx]

**Additional file 1**

**Standardised verbal instructions**

“I would like you to slump your shoulders forwards, while trying to place your chin on your chest. Now arch your back out like an angry cat. When you feel my finger pressing on your back, try to push your back out towards my finger. Remain sitting upright and try not to lean forwards or backwards”.
